# Environmental Stressors Suffered by Women with Gynecological Cancers in the Aftermath of Hurricanes Irma and María in Puerto Rico

**DOI:** 10.3390/ijerph182111183

**Published:** 2021-10-25

**Authors:** Pablo A. Méndez-Lázaro, Yanina M. Bernhardt, William A. Calo, Andrea M. Pacheco Díaz, Sandra I. García-Camacho, Mirza Rivera-Lugo, Edna Acosta-Pérez, Naydi Pérez, Ana P. Ortiz-Martínez

**Affiliations:** 1 Environmental Health Department, Graduate School of Public Health, Medical Sciences Campus, University of Puerto Rico, San Juan 00921, Puerto Rico; 2 Division of Cancer Control and Population Sciences, University of Puerto Rico Comprehensive Cancer Center, San Juan 00936, Puerto Rico; yanina.bernhardt@upr.edu (Y.M.B.); andrea.pacheco4@upr.edu (A.M.P.D.); sandra.garcia4@upr.edu (S.I.G.-C.); ana.ortiz7@upr.edu (A.P.O.-M.); 3 Department of Public Health Sciences, Penn State College of Medicine, Hershey, PA 17033, USA; wcalo@phs.psu.edu; 4 Center for Evaluation and Sociomedical Research, Graduate School of Public Health, Medical Sciences Campus, University of Puerto Rico, San Juan 00921, Puerto Rico; mirza.rivera@upr.edu (M.R.-L.); edna.acosta2@upr.edu (E.A.-P.); 5 Hispanic Alliance for Clinical and Translational Research, Medical Sciences Campus, University of Puerto Rico, San Juan 00921, Puerto Rico; naydi.perez@upr.edu; 6 Department of Biostatistics and Epidemiology, Graduate School of Public Health, Medical Sciences Campus, University of Puerto Rico, San Juan 00921, Puerto Rico

**Keywords:** extreme weather events, cancer patients, gynecological cancer, vulnerable populations, environmental stressors, Puerto Rico

## Abstract

Background: Hurricanes are the immediate ways that people experience climate impacts in the Caribbean. These events affect socio-ecological systems and lead to major disruptions in the healthcare system, having effects on health outcomes. In September 2017, Puerto Rico (PR) and the United States Virgin Islands (USVI) experienced one of the most catastrophic hurricane seasons in recent history (Hurricane Irma was a Category 5 and Hurricane María was a Category 4 when they hit PR). Objective: This study examines environmental stressors experienced by women with gynecologic (GYN) cancers from PR and USVI who received oncologic cancer care in PR, in the aftermath of the hurricanes. Methods: A descriptive qualitative study design was used to obtain rich information for understanding the context, barriers, knowledge, perspectives, risks, vulnerabilities, and attitudes associated to these hurricanes. We performed focus groups among GYN cancer patients (*n* = 24) and key-informant interviews (*n* = 21) among health-care providers and administrators. Interviews were conducted from December 2018–April 2019. Results: Environmental health stressors such as lack of water, heat and uncomfortable temperatures, air pollution (air quality), noise pollution, mosquitos, and rats ranked in the top concerns among cancer patients and key-informants. Conclusions: These findings are relevant to cancer patients, decision-makers, and health providers facing extreme events and disasters in the Caribbean. Identifying environmental secondary stressors and the most relevant cascading effects is useful for decision-makers so that they may address and mitigate the effects of hurricanes on public health and cancer care.

## 1. Introduction

Disasters such as powerful hurricanes have caused great catastrophes in the histories of many countries. These episodes usually have a marked impact on socio-ecological systems, such as infrastructure (i.e., supplies of drinking water, energy, transportation, and hospitals) and public health [1,2]. These extreme events are one of the immediate ways that people experience climate and weather impacts in the Caribbean. Low- and middle-income islands such as Puerto Rico and United States Virgin Islands (USVI) are particularly vulnerable to such events given their geographical location (exposed to hurricanes), the concentration of people, and crumbling infrastructure [3].

Aside from the direct effects that extreme events may bring on the population, there is a growing concern about the short- and long-term effects of weather on human health [4]. Research has shown that disasters and extreme weather events lead to major disruptions in the healthcare system and affect health outcomes [5,6,7]. Patients requiring essential services (e.g., cancer patients) are among the most vulnerable under extreme event scenarios [8,9]. Moreover, these extreme events are of concern in low- and middle- income countries with less prepared public health systems, and this can lead to disproportionately higher mortality rates.

In the context of climate change, more frequent and/or more intense extreme events are expected to affect vulnerable communities, human health (physical and mental), and well-being. The United Nations’ Sendai Framework for Disaster Reduction: 2015–2030 states that persons “with chronic diseases should be included in the design of policies and plans to manage their risks before, during, and after disasters [10]”. In particular, cancer patients are among the people with the greatest risk of death and complications after a disaster, as health care interruptions can negatively affect their health outcomes. Evidence suggests that for cancer patients, extreme events can limit their access to transportation, specialists, medications, and hospital services by limiting their access to cancer care and increasing their risk of premature death [6,11,12,13].

In September 2017, Puerto Rico experienced one of the most catastrophic hurricane seasons in recent history. On 13 September 2017, Hurricane Irma (Category 5) passed at 60 NM northeast from Puerto Rico, and on 20 September 2017, Hurricane María (Category 4) made landfall. Hurricanes Irma and Maria, coming so close together and with such tremendous force, resulted in much devastation and upheaval to so many lives and communities. Hurricane María is considered the strongest hurricane to make landfall in Puerto Rico since 1928, when the last category 5 hurricane was reported [14]. Hurricane María registered maximum winds of 135 kts, while the combined effect of the storm surge and tide produced levels of 3 to 9 ft above ground level. Nearly 38 in/24 h of rain occurred in Puerto Rico, proving to be the greatest 24-h rain intensity among all storms recorded on the island [15], with over 40,000 landslides [16] triggered by the storm.

After Hurricane María struck Puerto Rico, its population experienced major disruptions in essential services and many environmental stressors (e.g., water sanitation, contaminant exposure, vector borne diseases, food hygiene, carbon monoxide poisoning, and mold exposure). Environmental stress refers to how people respond to their environment’s physical, chemical, and biological features [17]. Stressors such as exposure to disasters, air and water pollution, climate change, or noise pollution can cause both short- and long- term impacts on human health and well-being.

Long before the widespread devastation caused by Hurricane María, Puerto Rico was in a dire social-ecological-technological situation, limiting the island’s capacity to be prepared, to respond, and to recover [18]. Evidence of this includes the following:

- Expensive essential infrastructure showing signs of advanced deterioration (e.g., water, energy, and transportation). According to recent reports, the total investment in infrastructure has been trending down for the last 18 years.
- One of the highest rates of unemployment in the US [19].
- The highest rates of the population living below poverty level in the US (43.1% persons in poverty) [19].

The impact of these hurricanes on treatment adherence and health outcomes of cancer patients has yet to be documented. Cancer is the second leading cause of death in Puerto Rico and the USVI [20]. This research is a first attempt to understand environmental stressors suffered by cancer patients in Puerto Rico and USVI in the aftermath of the 2017 Hurricane season, with a particular focus on female patients with gynecological (GYN) cancer. While these malignancies represent more than 15% of all cancers diagnosed in women in Puerto Rico [20], limited access to care existed prior to the hurricanes. During the study period, only five GYN oncologists provided services in Puerto Rico, and none in the USVI; many patients from USVI relocate for treatment to the continental United States and Puerto Rico. This research project examined psychosocial and environmental stressors experienced by women with GYN cancers from Puerto Rico and USVI who received oncologic cancer care in Puerto Rico in the aftermath of the hurricanes that contributed to unhealthy/unpleasant living conditions. Also, this research examined and described problems encountered by patients and as reported by clinics or organizations that provide care to this population.

## 2. Materials and Methods

### 2.1. Study Area

**Figure 1:**
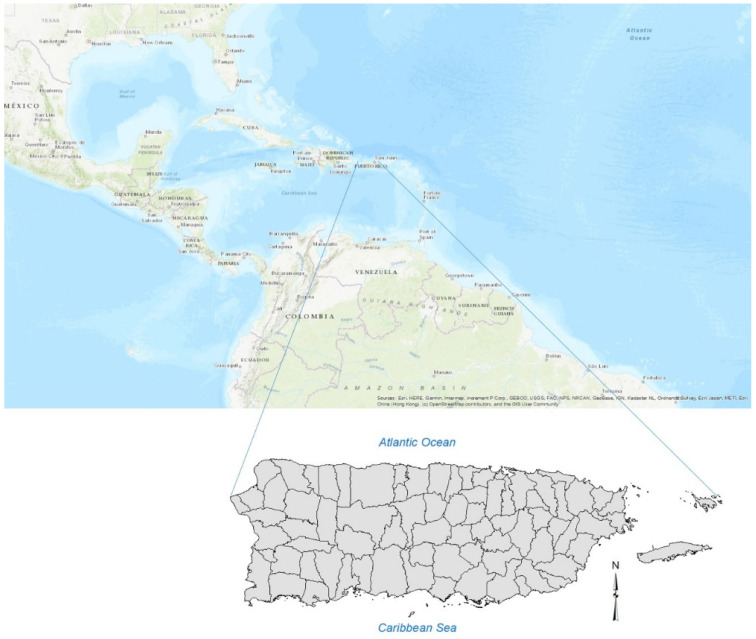
Puerto Rico base map.

Puerto Rico is an island located in the northern-central Caribbean Sea (17.92° N–18.52° N, 65.62° W–67.28° W), 8900 km2 in area and consisting of 78 municipalities (Figure 1). The island has a subtropical humid climate, with an annual average rainfall of ~1800 mm. Easterly trade winds prevail over the island for most of the year.

With a rapidly aging population and shrinking economy [21], Puerto Rico faces enormous environmental and public health challenges. Some of these issues are exacerbated by a higher incidence of severe droughts, urban and coastal floods, extreme heat episodes, and powerful tropical storms [22,23,24].

**Figure 2:**
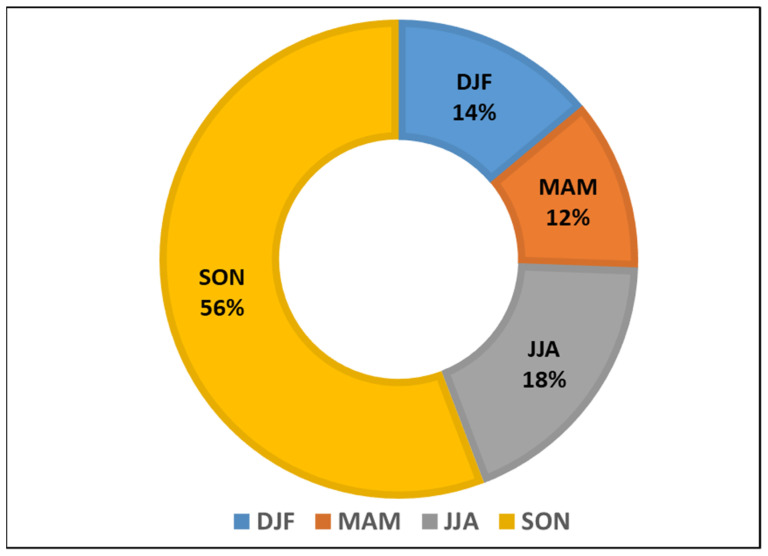
Disaster declarations per season (1956–2020): Winter (DJF: December-January-February; Spring (MAM: March-April-May); Summer (JJA: June-July-August); Falls (SON: September-October-November) Source: FEMA Disaster Declaration Summary 2020. Last Updated: 19 March 2020. Accessed 17 April 2020.

Because of its geographical location, the island suffers many tropical climate hazards each year (Figure 2). Before Hurricane María, Puerto Rico was declared a disaster zone on 43 occasions between 1956 and 2020 [25]. Most of the disaster declarations occurred in the fall (56%) or during the peak for hurricane season in the Caribbean (SON = September-October-November). During those months, the most frequent disaster declarations were for hurricanes (50%) and severe storms (25%).

### 2.2. Methodology

Although there are many different concepts of stress in the respective fields of medicine, psychology, and sociology, it is generally understood that stress is aversive for many people [26]. There are variations in vulnerability to environmental stressors that characterize human populations [27]. The vulnerability model of environmental stress identifies socioeconomic status, gender, race, and age as examples of demographic vulnerability to extreme events [28]. Because of the many definitions for primary and secondary stressors, for the purpose of this research, secondary stressors were defined as indirect problems that were triggered by the event but are not intrinsic to it, such as heat, noise pollution, air quality, and air pollution, mosquitos, and rats, among others. Primary stressors are those directly related to the event, such as strong winds, floods (urban, river, and coastal), and landslides. Primary stressors are those that are inherent in large accidents, disasters, and emergencies and that come directly from these events [29]. In some situations, people’s personal and social meaning derived from their experiences of an extreme event have more of a psychosocial influence on them than the event itself [29].

#### 2.2.1. Focus Groups GYN Cancer Patients: Study Design and Population

We conducted focus groups among women with GYN cancers. Exclusion/inclusion criteria were as follows. Only women 21 years and older from Puerto Rico and USVI, receiving oncologic care in Puerto Rico, with a diagnosis of GYN cancer, between September 2016 to September 2018, receiving services in any of the collaborating clinics in Puerto Rico, and who were physically and mentally competent to complete an interview, participated in these focus groups. The following gynecologic cancers were included based on the International Classification of Diseases for Oncology, 3rd edition (ICD-O-3) codes: vulva (C51), vagina (C52), cervix uteri (C53), corpus uteri (C54), uterus, NOS (C55), ovary (C56), or other unspecified female genital organs (C57) [30].

Recruitment. At the beginning of this research, we built an alliance with gynecology oncology clinics in Puerto Rico (San Juan, Mayagüez, and Ponce). Participating clinics helped the research team identify women as potential participants. A protocol was submitted to the Institutional Review Board (IRB) at the University of Puerto Rico-Medical Sciences Campus-Human Research Subjects Protection Office, and it was approved on 30 August 2018 (protocol #A1810418). The participating clinics were able to provide contact information for 31 women to the research team. Among those women, 24 (77%) agreed to participate in the focus groups.

Four focus groups were conducted between December 2018 and April 2019 in different locations of the island. Women with both public and private health insurance plans participated in the focus groups held in PR (*n* = 24). In addition, two participants from the USVI were interviewed individually by phone. The four focus groups were conducted in the maternal language of participants (Spanish), lasted from 90 to 120 min, and were audio-recorded. The two interviews with patients from USVI were conducted in English, lasting 60 min on average. For the purposes of the present work, we focus on stressors reported by GYN cancer patients.

#### 2.2.2. Key Informants (Health Providers and Administrators)

Between December 2018 and April 2019, we conducted in-depth key-informant interviews among health care professionals and administrators from Puerto Rico and USVI (*n* = 23). Our team interviewed individuals that provide services to women with GYN cancers in PR (oncologists, radiotherapists, nurses, administrators, and representatives from organizations that provide services to this population (including from USVI)), such as the PR Comprehensive Cancer Control Program, Cancer Control Coalition, the American Cancer Society and the PR Alliance for Chronic Disease Control. A semi-structured interview guide was developed based on a literature review [7,31,32] and the previous experience of the study researchers regarding the needs and concerns of providers and the health care system in the event of an extreme event and public health emergency preparedness.

These interviews took place in person or over the phone. They aimed to address problems encountered in their clinics and their organizations in the aftermath of the hurricanes (i.e., infrastructure, lack of power and water, electronic medical record), such as perceived psychosocial and environmental stressors and risks of their patients, difficulties in providing clinical services and strategies used to address them, the time that the clinic took to re-open, and recommendations for future preparedness efforts. For the present work, we focus on environmental stressors reported by key-informants.

#### 2.2.3. Qualitative Data Analysis

Focus group and interviews were transcribed verbatim by a bilingual transcriptionist and entered in Atlas.ti software (GMBH, Berlin, Germany) for analysis. All study team members received training on qualitative data analysis and coding using Atlas.ti software. A codebook was developed by the research team and collaborators of the Hispanic Alliance for Clinical and Translational Research. The codebook was tested with an initial set of transcripts and refined accordingly.

## 3. Results

### 3.1. General Qualitative Findings

GYN cancer patients: Participants’ mean age was 58 ± 13.2 years. The majority had an education higher than high school (73.1%), at least 46.2% had public health insurance coverage, and 42.3% were living below the poverty level (annual household income of $15,000 or lower). The sample comprised of women with diagnoses of ovarian (56%), corpus uteri (24%), cervical (16%), and vaginal (4%) cancers.

Key informants: Health providers (*n* = 11) and administrators (*n* = 12) participated in our interviews. Out of the 23 participants, 48% were medical doctors (36% hema-oncologists; 36% gyn-oncologists; 18% oncologists and 9% radio-oncologists).

### 3.2. Stressors in the Aftermath of Hurricane María

GYN cancer patients: Upon analyzing focus group discussions and interviews, we found that in the aftermath of Hurricane María (Category 4), many cancer patients experienced major disruptions in essential services (e.g., the availability of potable water and electric power, telecommunications, transportation) and environmental health issues (e.g., extreme heat, water sanitation, contaminant exposure, vector borne diseases, food hygiene, carbon monoxide poisoning and mold exposure).

The environmental stressors mentioned above ranked as one of the major concerns for both health providers and cancer patients in the aftermath of the events (Table 1). These identified stressors can interact and accumulate, influencing and prolonging stress, developing mental illness, and affecting well-being.

**Figure 3:**
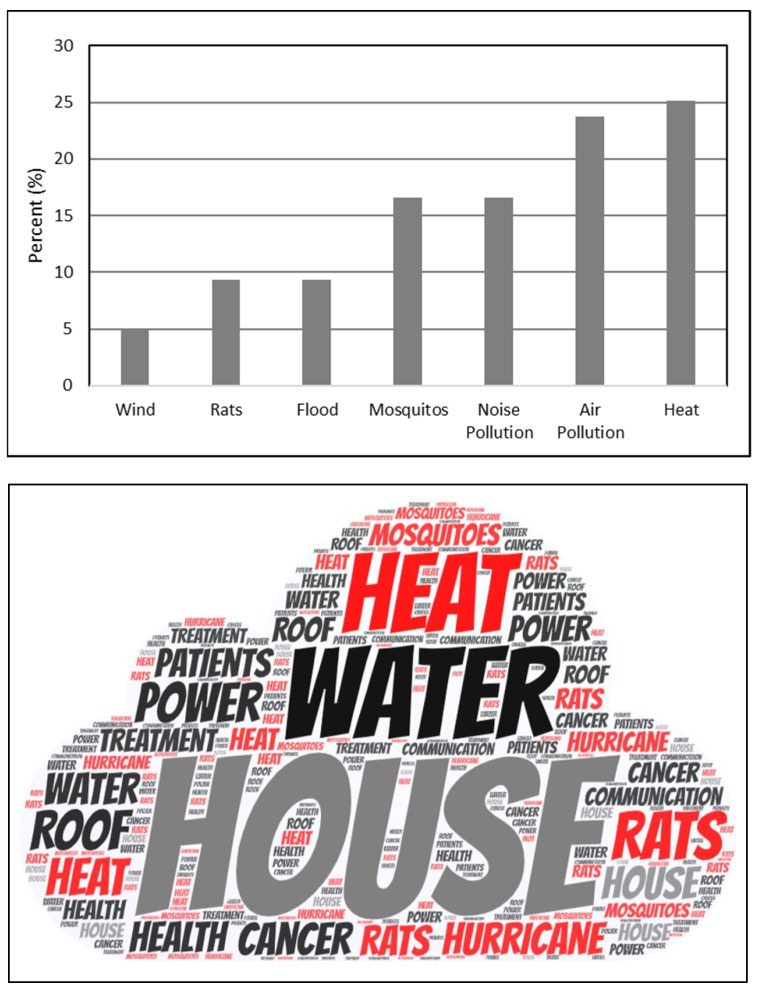
Upper Panel. Percentage of environmental stressors identified by cancer patients and health providers in the aftermath of Hurricane María and codified in transcripts. Lower panel: Wordcloud atlas of environmental stressors.

Within these stressors, environmental issues such as wind, water, heat and uncomfortable temperatures, air pollution (air quality), noise pollution, and mosquitos (Figure 3) ranked as the top concerns reported by cancer patients and health providers in the qualitative interviews. Among the environmental stressors reported by participants (*n* = 139 quotes), heat was mentioned 25% of the time as an environmental secondary stressor, ranking it in first place, followed by air pollution (23%) and noise pollution (16%) (Figure 3).

Table 2 includes quotes from participants in focus groups and key informant interviews. These quotes are intended to show (verbatim) the main environmental stressors suffered during the aftermath of Hurricane María; the heat was one of the main stressors reported by patients within their homes and by providers within the clinical setting.

## 4. Discussion

Surviving Hurricanes Irma and María were amongst the most recent extreme experiences faced by islanders who endured the natural disasters that devastated Puerto Rico and USVI during hurricane season 2017. Disasters such as Hurricanes Irma and María can have major, long-term impacts on people, families, and communities. Results showed that heat and uncomfortable temperatures, air pollution (air quality), noise pollution and mosquitoes were the top ranked environmental concerns reported in the aftermath of the hurricanes, followed by floods, rats and winds.

**Figure 4:**
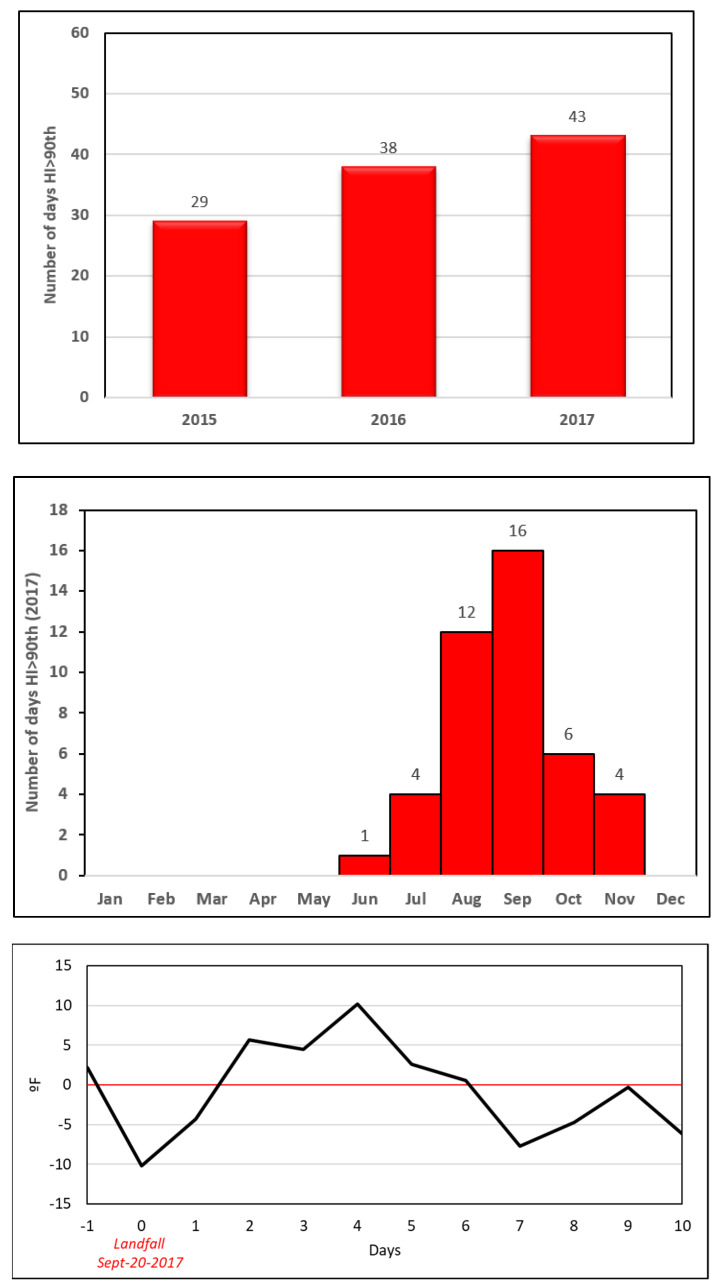
Upper panel: Number of days with heat index > 90th percentile (38 °C) per recent years. Middle panel: number of days with heat index > 90th percentile (38 °C) per months in 2017. Lower panel: days with heat index “above normal” after landfall. The climate data used in this study are derived from the Daymet version 2 dataset, a 1-km gridded product that provides daily values of precipitation and minimum and maximum temperature interpolated and extrapolated from the Global Historical Climatology daily surface observations [33].

Given that heat was among the major concerns for patients, to validate these observations, we examined a time series of daily air temperature (maximum and minimum) to evaluate variation. Our team analyzed air temperature and daily temperature ranges (DTR) for Puerto Rico using daily data from the Daymet version 2 dataset, ORNL DAAC, Oak Ridge, Tennessee, USA (Period 1 January 2015 to 31 December 2017). Daily Temperature Range (DTR) is the difference between the daily maximum and minimum temperatures. The climate data used were derived from the Daymet version 2 dataset [33]. During 2017, 43 days were above the 90th percentile (>38 °C/100 °F) for the heat index in the San Juan Metropolitan Area, with 16 of those occurrences found in September (Figure 4). Evaluated heat index data suggest that a heat episode occurred for five (5) consecutive days with temperatures “above normal” after landfall (>38 °C/100 °F). These results are consistent with cancer patients’ concerns. Cancer patients are more susceptible to heat episodes because, as explained by physicians, most of them can suffer from hot flashes [34,35,36].

Many researchers estimated excess mortality in the aftermath of Hurricane María [37]. These estimates vary among the different studies; however, most of them agreed on the causes for the excess mortality. Heart disease, septicemia, diabetes, respiratory problems, and cancer patients were among the first’s causes for “excess mortality” after Hurricane María in September 2017 [37,38]. Many factors could be associated with excess death, such as disruption of the electrical grid, communications, transportation, access to medical infrastructure, access to medicines, and healthcare services. Nevertheless, after interviewing cancer patients and health providers in this research, a fair assumption would be to consider the combination of stressors, including environmental factors promoting unhealthy and unpleasant conditions not only to cancer patients but also for many vulnerable communities in the aftermath of Hurricane María. Such conditions are solar radiation (in a warm-humid tropical environment), elevated air surface temperature + relative humidity (increasing heat index), floods, deforestation and canopy destruction, the lack of electricity and essential services, poor air quality (due to portable electric generators), vector-borne diseases and rodents, (rats and mosquitos [39]), potable water accessibility and water sanitation.

Disasters such as hurricanes could be considered hybrid forms of environmental stressors [29], resulting in primary stressors associated with the extreme event, such as winds, floods, storm surges, and landslides; while triggering environmental secondary stressors such as water pollution, air pollution, outbreaks, among others [40,41,42,43]. Often, these secondary environmental stressors are invisible and undetectable by humans and sometimes without the corresponding relevance in disaster management plans. The impact of recent hurricanes (e.g., Katrina, Sandy, Irma, and Maria) demonstrates failures in built infrastructure, the inadequacy of institutions, the lack of resources, and dire information systems. These failures could be triggering a worst-case context for environmental stress resulting in technological failure, ecological contamination, and toxic exposure for humans. As an example, Hurricane Katrina was not only a massive meteorological phenomenon but also a massive contamination event, causing the release of millions of gallons of oil into the environment [42,43]. During the aftermath of Hurricane María in Puerto Rico, a significant increase in air pollution (e.g., SO_2_) and a negative impact on water quality were documented [44]. In fact, researchers provided evidence for drinking water quality problems in Puerto Rico in the aftermath of Hurricane María, suggesting that metals (i.e., arsenic) and PFOA were the top ranked pollutants in communities of the northern region of the island [45].

However, this is the first time suggesting that heat and uncomfortable temperatures were among the main environmental concerns reported by GYN cancer patients in the aftermath of the hurricanes. There is evidence suggesting that higher frequency, duration, and intensity of extreme heat episodes are triggering public health issues on the island [24,46].

With urbanization, landscape transformation has enhanced the urban heat island (UHI) effect in the San Juan metropolitan area of Puerto Rico, increasing urban temperatures [47]. The hottest areas corresponded to high-density urbanized areas with little Forest and little green infrastructure. Green infrastructure (e.g., urban vegetation, urban forest), and healthy ecosystems provide unique ecological services such as reducing runoff, energy-cooling savings, regulating temperatures [48], and oxygen production [49,50,51]. However, a rapid assessment estimated that all urban areas in Puerto Rico suffered tree cover loss (e.g., San Juan 24.8%; Ponce 6.2%; Mayagüez 4.2%) [48]. Hurricane María also impacted forests, terrestrial ecosystems, tripling stem breaks, and doubled tree mortality all over the island [48,49]. Therefore, we can hypothesize that the combination of tree mortality, loss of green infrastructure, incident solar radiation (in a warm-humid tropical environment) and the lack of electricity could be factors triggering unpleasant temperatures for cancer patients in the aftermath of Hurricane María.

**Figure 5:**
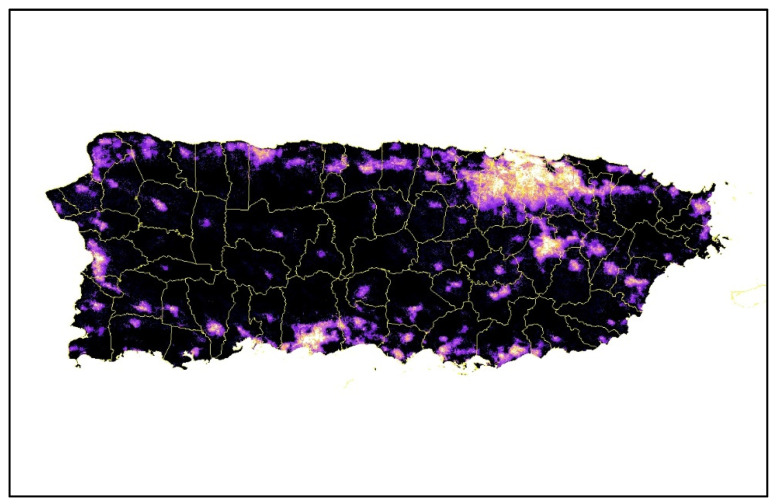

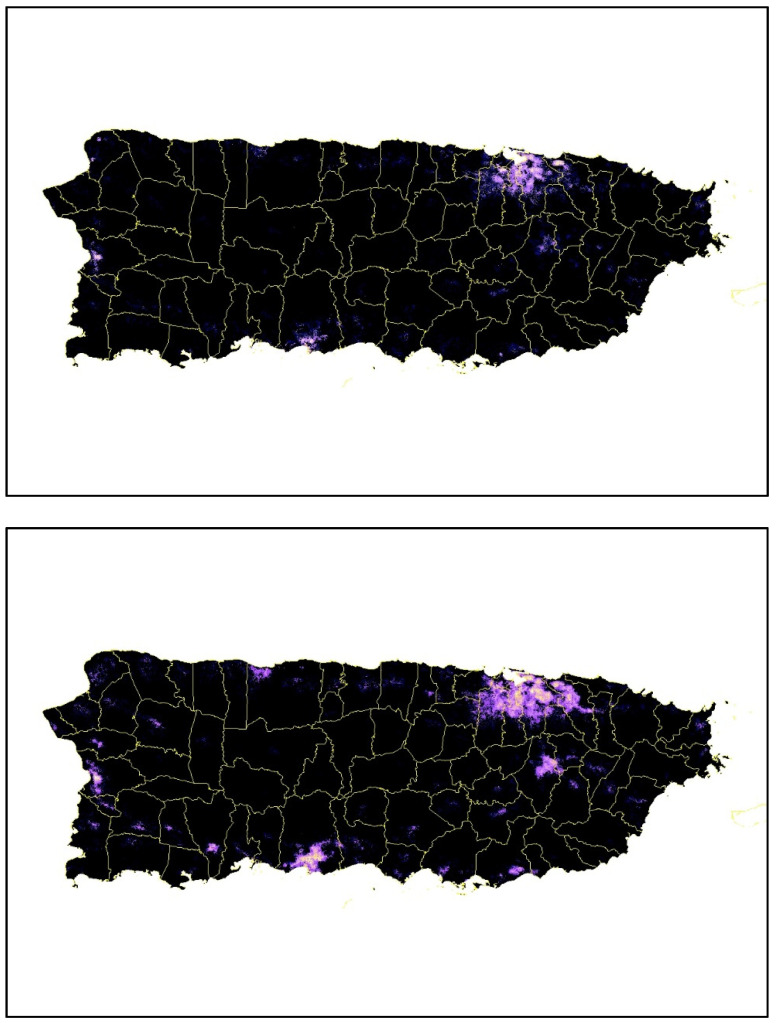
Upper panel: Lights before the storm; center panel: Lights after the storm (20 September–20 November 2017), lower panel: Four months after the storm (21 November 2017–2020 January 2018). Source [55].

Some heat-humidity impacts can be avoided through acclimation and behavioral adaptation [52,53]. However, there exists an upper limit for survivability under sustained exposure and extreme conditions [53,54], even with idealized conditions and available essential services (such as electricity, perfect health, full shade, and water). Hurricane María caused the longest electricity blackout in the history of the United States (Figure 5); 41% of the lengthy power outages post-María occurred in rural areas, and 29% of the lengthy power outages occurred in urban areas [55]. The impact on the electric grid left millions of residents without power and unable to mitigate extreme temperatures by using air conditioners, fans or similar equipment (e.g., acclimation technological-adaptation options).

The deadly heat events already experienced in recent decades are indicative of the continuing trend towards increasingly extreme humid heat representing a major societal challenge for the coming decades in Puerto Rico and other Caribbean island nations. To our understanding, this research is the first attempt to understand environmental secondary stressors suffered by women with gynecological cancer in Puerto Rico in the aftermath of Hurricanes Irma and María. These research findings are relevant to cancer patients facing extreme weather events and disasters in the Caribbean. The method’s design allowed us to obtain relevant and rich information in different regions of the island, which can be considered in other parts of the world facing similar disasters. One limitation of this manuscript is that we analyzed qualitative information from only one chronic disease in Puerto Rico. Our research team acknowledges there are many other vulnerable patients with prevalent chronic health conditions that might had their treatments disrupted by the disaster, potentially suffering similar stressors as the participants in this study.

## 5. Conclusions

Extreme weather- and climate-related disasters have social-ecological and technological dimensions producing extreme forms of environmental stress that threaten public health and the social well-being of human populations. To our knowledge, this is one of the few studies trying to understand the context, barriers, knowledge, health providers’ perspective, risks and vulnerabilities, perceptions, patient profiles, and attitudes experienced by women with gynecological cancer in the aftermath of Hurricanes Irma and María.

Despite the widespread devastation caused by Hurricane María, the island of Puerto Rico was in a dire situation before the hurricane struck the island, limiting the island’s capacity to prepare, respond and recover. According to the fourth National Climate Assessment for the Caribbean Region 2018 [22], “High levels of exposure and sensitivity to risk in the U.S. Caribbean region are compounded by a low level of adaptive capacity, due in part to the high costs of mitigation and adaptation measures relative to the region’s gross domestic product”.

As we write this manuscript, the state and federal governments were allocating budget resources and making decisions to rebuild Puerto Rico. However, we argue that we must do more than rebuild (business as usual); we must transform the infrastructural, social, and public health conditions that made us vulnerable to these catastrophic events in the first place. The following years will be critical to ensure that rebuilding efforts are inclusive of alternative, transformative visions and perspectives, and most importantly, the needs of the people and ecosystems that will be most vulnerable to future extreme events. In addition, our study findings support the need to strengthen resilience and adaptive capacity to disasters in cancer plans [56]. Further research should emphasize a better understanding of those compounds and cascading effects triggering secondary environmental stress affecting public health. Finally, future disaster management plans should anticipate and address chronic care needs and secondary stressors with the timely reestablishment of primary care services, access to medications, and means to address financial and structural barriers to treatment [56].

Additionally, the knowledge gained through this qualitative study helped guide the execution and demonstrates the need for the ongoing quantitative study being performed by our research team, in which a retrospective cohort study is assessing the impact of both environmental and psychosocial stressors on health outcomes and use of health care services among GYN cancer patients in the aftermath of the hurricanes.

## Figures and Tables

**Table 1:** Code L5: Primary and secondary environmental stressors.

Code	Code Definition	Absolute Frequency		
		Key Informants (Health Providers and Administrators	Focus Groups (GYN Cancer Patients)	Total
L5.B Secondary Stressor	Stressors not inherent to the meteorological phenomenon. Secondary stressors could be those occurring during the aftermath of a climate-related disaster. Attributable or partially attributable to the disaster.	86	73	159
L5.D Physical/Environment Psychosocial Stressor	Experienced by women after the hurricanes. Evacuations, being displaced outside the home, transportation difficulties, time without electricity, telecommunications, water, availability of a power plant, damage to the home, access to materials and supplies, food, distance from the home to the clinic, and security. It is a person’s reaction to a specific situation in which a set of environmental variables are present whose disposition and intensity make them perceived as aversive.	32	53	85
L5.E Psychosocial Stressor of Health Care Systems	Experienced by women after the hurricanes. Lack of available health services.	48	32	80

**Table 2:** Environmental stressors reported by GYN Cancer patients and Key-Informants.

Focus Groups: Cancer Patients		
Code ID	Original Version (Spanish)	English Version
D 22: 26 Transcription, Focus Group No. 3	“*…el calor estaba chispeante, pero tu tienes que pensar, que tu no puedes dormir bajo 4 o 5 paredes de cemento, porque el calor no hay manera de sacarlo. Así que tienes que tener la alternativa de dormir afuera. Yo dormí en la terraza!*”	“…the heat was sparkling, but you have to think that you cannot sleep under four or five concrete walls because there is no way to get rid of the heat. Therefore, you have to have the alternative of sleeping outside. I slept on the terrace!”
D 21: 24 Transcription, Focus Group No. 1	“*Yo lloraba, hasta cuándo no vamos a tener luz, era bien frustrante! Y chequeándola a ver si estaba respirando, porque mi miedo era que por tanta calor le diera un bajón de azúcar y uno durmiendo. Y entonces yo la tocaba y a veces no la veía y era como que estaba respirando y yo ok. Pero fue bien frustrante.*”	“I was crying, until when we will not have electricity, it was very frustrating! And checking her to see if she was breathing because my fear was that because of so much heat, she could have a low sugar episode and me sleeping. And then I would touch her, and sometimes I didn’t see her, and it was like she was breathing, and I was ok. But it was very frustrating.”
D 29: 25 Transcription, Focus Group No. 2:	“*Pues si yo no tenía planta, claro que había calor! De hecho fíjate, llegaba un momento en donde uno…no tengo malos recuerdos de esa…O sea lo único que malo es lo de las plantas, (todos: el olor) las emisiones, es lo único, pero si a veces de noche uno no podía dormir por el calor, es verdad.*”	“Well, if I didn’t have a generator, of course, it was hot! In fact, look, there came a time when one … I don’t have bad memories of that … I mean the only bad thing is the thing of the generators, (all: the smell) the emissions, it’s the only thing, but yes, sometimes at night one could not sleep because of the heat, it is true.”
D 4: 27 Transcription, Focus Group No. 4	“*Los mosquitos y el calor* *-fue bien difícil* *-yo no dormía casi*”	“Mosquitoes and heat -It was very difficult -I hardly slept”
D 29: 25 Transcription, Focus Group No. 2	“*Pienso que le hubiese hecho caso a mi padre, de haber comprado la planta a tiempo, antes del huracán- antes del huracán! Y ese revolú de estar buscando agua, de que mi vecina llegara para conectar la nevera y más que nada las emisiones. Yo diría que eso fue lo más estrés que a mí me dio, siempre estaba viendo, y mi hijo; “por dónde viene el viento?, mira a ver, identifica. Okey, cierra estas ventanas! Después vuelve y abre, okey ahora vamos a cerrar las otras.” No dormía, como usted preguntó. No se dormía, por el calor. El calor era una cosa…yo no se si ustedes se acuerdan que hubo par de días que el calor, a menos que bueno si usted estaba durmiendo con aire pues* *(rien)* *- pero el calor fue una cosa bien intensa, bien intensa, yo vivo en Caguas*”	“I think I would have listened to my father, of having had the generator on time, before the hurricane—before the hurricane! And that scramble of being looking for water, that my neighbor came to connect the refrigerator and more than anything the emissions. I would say that was the most stress that gave me, I was always watching, and my son; “Where does the wind come from? Look to see, identify. Okay, close these windows! Then come back and open, okay, now we are going to close the others. “ I wasn’t sleeping, as you asked. You couldn’t sleep because of the heat. The heat was one thing … I don’t know if you remember that there were a couple of days in which the heat, unless well if you were sleeping with air conditioning, then (group laughs) - but the heat was a very intense, very intense thing, I live in Caguas”
Key Informant Interviews		
D 19: 18 Interview Transcription No. 18	“*Acuérdate que los pacientes que están (en tratamiento) de quimioterapia sufren mucho de calores, y hot flashes, muchas veces sudan de más (mucho), y están enfermos. Algunos vomitan, y al no tener la luz (electricidad) ni un abanico para refrescarse cuando uno se siente mal. (Tener) Hielo para refrescarse por dentro, eso es (de) lo que más se quejaron.*”	“Remember that patients who are undergoing chemotherapy suffer a lot from heats and hot flashes; they sweat more (often), and they are sick. Some of them vomit, and not even having (electricity) a fan to cool off when you feel sick is not comfortable. Not having Ice to cool your organism inside, that’s what patients complained the most.”

## Data Availability

The environmental data presented in this study are openly available in: Daily Surface Weather Data on a 1-km Grid for North America, Version 4. Available online: https://daac.ornl.gov/cgi-bin/dsviewer.pl?ds_id=1840 (accessed on 8 May 2020). The qualitative dataset is available from the corresponding author upon reasonable collaboration request.
